# Supplementary material for: Pyruvate Dehydrogenase Kinase 1 inhibition mediated oxidative phosphorylation enhancement in cartilage promotes osteoarthritis progression
Source: BMC Musculoskelet Disord. 2023 Jul 20;24:597. doi: 10.1186/s12891-023-06585-6 (PMC10357736; doi:10.1186/s12891-023-06585-6)
Supplement: Supplementary file 1 — Additional file 1. [file 12891_2023_6585_MOESM1_ESM.zip › BP.Gsea.1653623667859/GOBP_NEGATIVE_REGULATION_OF_MITOCHONDRION_ORGANIZATION.html]

Details for gene set GOBP\_NEGATIVE\_REGULATION\_OF\_MITOCHONDRION\_ORGANIZATION[GSEA]

|  || Dataset | 55457\_collapsed\_to\_symbols.cls.cls #OA\_versus\_normal.cls.cls #OA\_versus\_normal\_repos |
| Phenotype | cls.cls#OA\_versus\_normal\_repos |
| Upregulated in class | OA |
| GeneSet | GOBP\_NEGATIVE\_REGULATION\_OF\_MITOCHONDRION\_ORGANIZATION |
| Enrichment Score (ES) | 0.47568336 |
| Normalized Enrichment Score (NES) | 1.8795229 |
| Nominal p-value | 0.0 |
| FDR q-value | 0.04659875 |
| FWER p-Value | 0.82 |
Table: GSEA Results Summary

  

Fig 1: Enrichment plot: GOBP\_NEGATIVE\_REGULATION\_OF\_MITOCHONDRION\_ORGANIZATION      
 Profile of the Running ES Score & Positions of GeneSet Members on the Rank Ordered List

  

| SYMBOL | TITLE | RANK IN GENE LIST | RANK METRIC SCORE | RUNNING ES | CORE ENRICHMENT || 1 | PARL | presenilin associated rhomboid like [Source:HGNC Symbol;Acc:HGNC:18253] | 14 | 1.156 | 0.0997 | Yes |
| 2 | PSMD10 | "proteasome 26S subunit, non-ATPase 10 [Source:HGNC Symbol;Acc:HGNC:9555]" | 58 | 0.976 | 0.1815 | Yes |
| 3 | TMEM14A | transmembrane protein 14A [Source:HGNC Symbol;Acc:HGNC:21076] | 161 | 0.848 | 0.2474 | Yes |
| 4 | GHITM | growth hormone inducible transmembrane protein [Source:HGNC Symbol;Acc:HGNC:17281] | 213 | 0.809 | 0.3140 | Yes |
| 5 | TSPO | translocator protein [Source:HGNC Symbol;Acc:HGNC:1158] | 692 | 0.595 | 0.3288 | Yes |
| 6 | AKT1 | AKT serine/threonine kinase 1 [Source:HGNC Symbol;Acc:HGNC:391] | 1192 | 0.478 | 0.3317 | Yes |
| 7 | MUL1 | mitochondrial E3 ubiquitin protein ligase 1 [Source:HGNC Symbol;Acc:HGNC:25762] | 1610 | 0.412 | 0.3351 | Yes |
| 8 | PRKN | parkin RBR E3 ubiquitin protein ligase [Source:HGNC Symbol;Acc:HGNC:8607] | 1972 | 0.364 | 0.3388 | Yes |
| 9 | CLU | clusterin [Source:HGNC Symbol;Acc:HGNC:2095] | 2083 | 0.352 | 0.3609 | Yes |
| 10 | PINK1 | PTEN induced kinase 1 [Source:HGNC Symbol;Acc:HGNC:14581] | 2097 | 0.350 | 0.3904 | Yes |
| 11 | TRIAP1 | TP53 regulated inhibitor of apoptosis 1 [Source:HGNC Symbol;Acc:HGNC:26937] | 2163 | 0.342 | 0.4152 | Yes |
| 12 | IGF1 | insulin like growth factor 1 [Source:HGNC Symbol;Acc:HGNC:5464] | 2286 | 0.328 | 0.4344 | Yes |
| 13 | HGF | hepatocyte growth factor [Source:HGNC Symbol;Acc:HGNC:4893] | 2547 | 0.297 | 0.4401 | Yes |
| 14 | TIGAR | TP53 induced glycolysis regulatory phosphatase [Source:HGNC Symbol;Acc:HGNC:1185] | 2580 | 0.293 | 0.4631 | Yes |
| 15 | ACAA2 | acetyl-CoA acyltransferase 2 [Source:HGNC Symbol;Acc:HGNC:83] | 2779 | 0.272 | 0.4715 | Yes |
| 16 | RNF41 | ring finger protein 41 [Source:HGNC Symbol;Acc:HGNC:18401] | 3005 | 0.249 | 0.4757 | Yes |
| 17 | MAPT | microtubule associated protein tau [Source:HGNC Symbol;Acc:HGNC:6893] | 3976 | 0.158 | 0.4140 | No |
| 18 | SLC35F6 | solute carrier family 35 member F6 [Source:HGNC Symbol;Acc:HGNC:26055] | 4154 | 0.142 | 0.4126 | No |
| 19 | VAT1 | vesicle amine transport 1 [Source:HGNC Symbol;Acc:HGNC:16919] | 4779 | 0.088 | 0.3718 | No |
| 20 | BNIP3 | BCL2 interacting protein 3 [Source:HGNC Symbol;Acc:HGNC:1084] | 5501 | 0.032 | 0.3186 | No |
| 21 | TP53 | tumor protein p53 [Source:HGNC Symbol;Acc:HGNC:11998] | 5511 | 0.032 | 0.3206 | No |
| 22 | BCL2L2 | BCL2 like 2 [Source:HGNC Symbol;Acc:HGNC:995] | 5643 | 0.020 | 0.3122 | No |
| 23 | PPARG | peroxisome proliferator activated receptor gamma [Source:HGNC Symbol;Acc:HGNC:9236] | 6006 | -0.005 | 0.2845 | No |
| 24 | HUWE1 | "HECT, UBA and WWE domain containing E3 ubiquitin protein ligase 1 [Source:HGNC Symbol;Acc:HGNC:30892]" | 6558 | -0.046 | 0.2457 | No |
| 25 | BCL2L1 | BCL2 like 1 [Source:HGNC Symbol;Acc:HGNC:992] | 6698 | -0.055 | 0.2397 | No |
| 26 | FZD9 | frizzled class receptor 9 [Source:HGNC Symbol;Acc:HGNC:4047] | 6812 | -0.064 | 0.2365 | No |
| 27 | TSC2 | TSC complex subunit 2 [Source:HGNC Symbol;Acc:HGNC:12363] | 6946 | -0.075 | 0.2327 | No |
| 28 | AVP | arginine vasopressin [Source:HGNC Symbol;Acc:HGNC:894] | 7201 | -0.096 | 0.2213 | No |
| 29 | ARRB2 | arrestin beta 2 [Source:HGNC Symbol;Acc:HGNC:712] | 7255 | -0.100 | 0.2259 | No |
| 30 | NOL3 | nucleolar protein 3 [Source:HGNC Symbol;Acc:HGNC:7869] | 7545 | -0.122 | 0.2141 | No |
| 31 | HSPA1A | heat shock protein family A (Hsp70) member 1A [Source:HGNC Symbol;Acc:HGNC:5232] | 7876 | -0.146 | 0.2012 | No |
| 32 | SLC25A5 | solute carrier family 25 member 5 [Source:HGNC Symbol;Acc:HGNC:10991] | 7877 | -0.146 | 0.2139 | No |
| 33 | IAPP | islet amyloid polypeptide [Source:HGNC Symbol;Acc:HGNC:5329] | 7976 | -0.153 | 0.2197 | No |
| 34 | FXN | frataxin [Source:HGNC Symbol;Acc:HGNC:3951] | 8057 | -0.160 | 0.2274 | No |
| 35 | GCLC | glutamate-cysteine ligase catalytic subunit [Source:HGNC Symbol;Acc:HGNC:4311] | 8534 | -0.196 | 0.2075 | No |
| 36 | OPA1 | OPA1 mitochondrial dynamin like GTPase [Source:HGNC Symbol;Acc:HGNC:8140] | 8655 | -0.205 | 0.2160 | No |
| 37 | FLCN | folliculin [Source:HGNC Symbol;Acc:HGNC:27310] | 8848 | -0.221 | 0.2203 | No |
| 38 | PPIF | peptidylprolyl isomerase F [Source:HGNC Symbol;Acc:HGNC:9259] | 9999 | -0.321 | 0.1589 | No |
| 39 | TFRC | transferrin receptor [Source:HGNC Symbol;Acc:HGNC:11763] | 10184 | -0.339 | 0.1742 | No |
| 40 | LMNA | lamin A/C [Source:HGNC Symbol;Acc:HGNC:6636] | 10946 | -0.424 | 0.1519 | No |
Table: GSEA details [plain text format]

  

Fig 2: GOBP\_NEGATIVE\_REGULATION\_OF\_MITOCHONDRION\_ORGANIZATION      
 Blue-Pink O' Gram in the Space of the Analyzed GeneSet

  

Fig 3: GOBP\_NEGATIVE\_REGULATION\_OF\_MITOCHONDRION\_ORGANIZATION: Random ES distribution      
 Gene set null distribution of ES for **GOBP\_NEGATIVE\_REGULATION\_OF\_MITOCHONDRION\_ORGANIZATION**
